# Refraining from closed reduction of displaced distal radius fractures in the emergency department—in short: the RECORDED trial

**DOI:** 10.1186/s13063-024-08118-5

**Published:** 2024-05-06

**Authors:** B. M. Derksen, P. A. Jawahier, O. Wijers, S. P. Knops, M. R. de Vries, C. C. Drijfhout van Hooff, M. H. J. Verhofstad, N. W. L. Schep

**Affiliations:** 1 Department of Trauma Surgery, Maasstad Hospital, Rotterdam, the Netherlands; 2 Department of Trauma Surgery, Franciscus Gasthuis and Vlietland, Rotterdam, the Netherlands; 3 Department of Trauma Surgery, Ikazia Hospital, Rotterdam, the Netherlands; 4 Department of Trauma Surgery, IJsselland Hospital Rotterdam, Capelle Aan Den IJssel, the Netherlands; 5 Department of Trauma Surgery, Albert Schweitzer Hospital, Dordrecht, the Netherlands; 6 Department of Trauma Surgery, Erasmus Medical Centre, Rotterdam, the Netherlands

**Keywords:** Distal radius fracture, Closed reduction, Surgery, Cost-effectiveness

## Abstract

**Background:**

With roughly 45,000 adult patients each year, distal radius fractures are one of the most common fractures in the emergency department. Approximately 60% of all these fractures are displaced and require surgery. The current guidelines advise to perform closed reduction of these fractures awaiting surgery, as it may lead to post-reduction pain relief and release tension of the surrounding neurovascular structures. Recent studies have shown that successful reduction does not warrant conservative treatment, while patients find it painful or even traumatizing. The aim of this study is to determine whether closed reduction can be safely abandoned in these patients.

**Methods:**

In this multicenter randomized clinical trial, we will randomize between closed reduction followed by plaster casting and only plaster casting. Patients aged 18 to 75 years, presenting at the emergency department with a displaced distal radial fracture and requiring surgery according to the attending surgeon, are eligible for inclusion. Primary outcome is pain assessed with daily VAS scores from the visit to the emergency department until surgery. Secondary outcomes are function assessed by PRWHE, length of stay at the emergency department, length of surgery, return to work, patient satisfaction, and complications. A total of 134 patients will be included in this study with follow-up of 1 year.

**Discussion:**

If our study shows that patients who did not receive closed reduction experience no significant drawbacks, we might be able to reorganize the initial care for distal radial fractures in the emergency department. If surgery is warranted, the patient can be sent home with a plaster cast to await the call for admission, decreasing the time spend in the emergency room drastically.

**Trial registration:**

This trial was registered on January 27, 2023.

## Administrative information

Note: the numbers in curly brackets in this protocol refer to SPIRIT checklist item numbers. The order of the items has been modified to group similar items (see http://www.equator-network.org/reporting-guidelines/spirit-2013-statement-defining-standard-protocol-items-for-clinical-trials/).

**Table:** 

Title {1}	Refraining from closed reduction of dislocated distal radius fractures in the emergency department: the RECORDED-trial
Trial registration {2a and 2b}.	NCT06046404 on ClinicalTrials.gov.
Protocol version {3}	1.0.
Funding {4}	.This research was largely funded by the BeterKeten research grant.
Author details {5a}	B.M. Derksen MD1, P.A. Jawahier MD1, O. Wijers MD PhD2, S.P. Knops MD PhD3, M.R. de Vries MD PhD4, C.C. Drijfhout van Hooff MD5, M.H.J. Verhofstad MD PhD6, N.W.L. Schep MD PhD1 1Department of trauma surgery, Maasstad Hospital, Rotterdam 2Department of trauma surgery, Franciscus Gasthuis & Vlietland 3Department of trauma surgery, Ikazia Hospital, Rotterdam 4Department of trauma surgery, IJsselland Hospital Rotterdam 5Department of trauma surgery, Albert Schweitzer Hospital 6Department of trauma surgery, Erasmus Medical Centre, Rotterdam
Name and contact information for the trial sponsor {5b}	Department of Trauma and Hand Surgery, Maasstad Hospital Rotterdam, Maasstadweg 21, 3079 DZ, Rotterdam, The Netherlands
Role of sponsor {5c}	This trial is an investigator initiated clinical trial.

## Introduction

### Background and rationale {6a}

Each year, almost 26,000 adults are treated for a displaced distal radius fracture (DRF) in the Netherlands, making it one of the most common fractures in the ED [1–4]. According to a Swedish study, approximately 60% (= 16,000) of all DRFs are displaced [5]. The majority of all displaced DRFs are treated with osteosynthesis. This surgery is semi-acute planned operative care, mostly within the first week after trauma.

The Dutch DRF guidelines advise to perform closed reduction (CR) of displaced DRFs awaiting surgery at the ED, because it may lead to post-reduction pain relief, and a successful reduction may warrant conservative treatment [6]. Techniques used for CR of DRFs are manual traction and finger-trap traction [7]. Both procedures are often painful despite injection of a local anesthetic between the fracture fragments [8–10]. Sometimes, the patient is even sedated (as well). Besides the physical discomfort associated with CR, patients may experience CR as a traumatizing event as well. This questions how relevant CR at the emergency department really is. The same guideline also acknowledges that evidence supporting the advice to perform a CR is lacking [11, 12]. Meanwhile, CR is a painful, costly, time-consuming procedure, often requiring anesthesia and most surgeons experience a substantial re-dislocation at surgery.

Furthermore, the guideline suggests that good fracture alignment justifies conservative treatment. This recommendation is based on three rather outdated randomized studies comparing cast immobilization with either external fixation of the wrist or percutaneous pin fixation [13–15]. However, both surgical techniques have been largely replaced by internal plate fixation. Recent European randomized controlled trials have shown that in displaced DRFs, despite acceptable fracture reduction, volar plating is superior to cast immobilization in terms of functional outcome, quality of life, and prevention of secondary dislocation [16–18]. This was also concluded in a large meta-analysis of 23 trials including 2254 patients, published last year [19]. Opposed to the current but rather old guideline, strong recent evidence suggests that operative treatment is preferred over conservative treatment in adult patients with displaced DRFs. A trend towards more operative treatments is expected and makes our research question more relevant.

### Objectives {7}

The aim of this multicenter, randomized clinical trial is to determine if CR in patients with a displaced DRF can be safely abandoned before the plaster cast, as a bridge to surgery, is applied.

### Trial design {8}

This trial is designed as a non-inferiority multicenter cluster randomized trial. Participants will be equally distributed between the two treatment arms.

## Methods: participants, interventions, and outcomes

### Study setting {9}

The study will be situated at six public hospitals in and round the city of Rotterdam. All participating hospitals are part of the BeterKeten foundation that provided the research grant. The group consists of both rural and academic- and city-centered hospitals.

### Eligibility criteria {10}

All patients aged between 18 and 75 years with a displaced distal radius fracture requiring surgery will be approached. A DRF is considered displaced with an indication for surgery if one of the following criteria is met:

- Articular gap or step-off ≥ 2 mm. The intra-articular gap is defined as the maximal distance between fracture fragments parallel to the articular surface. The intra-articular step-off is defined as the maximal distance between fracture fragments perpendicular to the articular surface [16]
- Carpal alignment > 5 mm. The carpal alignment represents the length of a perpendicular line between a line along the inner rim of the volar cortex of the radius and the center of the capitate [18]
- Dorsal angulation > 10°, volar angulation > 20°. The dorsal/volar angulation represents the angle between a line that connects the dorsal and volar rim of the distal radius and a line perpendicular to the longitudinal axis of the radius [20]
- Radial height loss > 3 mm. Also known as the radial length, defined as the distance between two lines, both perpendicular to the long axis of the radius. One line goes through the tip of the radial styloid, and the second goes through the most distal point of the ulnar head [21]
- Radial inclination < 15°. The radial inclination is the angle between two lines. One line connects the radial styloid process with a point on the ulnar aspect of the distal radius, in the center between the dorsal and volar rim. The second line is directed perpendicularly to the longitudinal axis of the radius [20]
- Coronal plane translation. This term is used to describe radial displacement of the distal fragment. Radial translation of the distal fragment might be associated with DRUJ instability due to lack of tension on the distal oblique bundle (the most distal part of the distal interosseous membrane) and the pronator quadratus. Coronal plane translation can be measured by drawing a line on the ulnar side of the radius which intersects the lunate. The point of intersection within the lunate is evaluated by drawing a second line along the transverse width of the lunate parallel with the distal joint. In a normal situation, the lines should bisect at 50% [22, 23]
- Incongruent DRUJ: the ulnar side of the distal radius and radial side of the ulna should converge in the form of a Gothic arc. This arc should not be interrupted

The criteria for exclusion are:

- Patients younger than 18 years or older than 76 years old
- Acute limb threatening ischemia, defined as: “any sudden decrease in limb perfusion causing a potential threat to limb viability” [24]
- Skin tenting or symptoms that suggest an impending open fracture
- Acute symptoms of median nerve compression, such as paresthesia in digit 1–3, or extending to the entire hand, pain, hand weakness, and cramps in the hand [25]
- Other fractures in the same or other upper extremity which require a separate treatment
- Previous distal radius fracture in the ipsilateral wrist < 3 months
- Multiple trauma patients (Injury Severity Score (ISS) ≥ 16)
- Patients with impaired wrist function due to previous injuries, bone disorders, or neurological disorders
- Insufficient comprehension of the Dutch language to understand a rehabilitation program and other treatment information as judged by the attending physician
- Inability to complete the study period (i.e., patients from abroad who will have surgery outside one of the participating hospitals)

### Who will take informed consent? {26a}

Informed consent will be obtained by the attending physician at the emergency department. All physicians will be either trained in the protocol or supervised by someone who is.

### Additional consent provisions for collection and use of participant data and biological specimens {26b}

There is a separated section on the informed consent form where participants can check a box to give consent for the use of date for ancillary studies. This trial does not involve collecting biological specimens for storage.

## Interventions

### Explanation for the choice of comparators {6b}

This trial will compare closed reduction prior to surgery in displaced distal radial fractures to no closed reduction prior to surgery. Currently, the Dutch guidelines state there is a knowledge gap concerning the efficacy of closed reduction prior to surgery. Therefore, either outcome of this study would lead to an update of said guideline. In daily practice, both treatments are used at the moment of writing this protocol.

### Intervention description {11a}

Closed reduction of distal radial fractures is performed with axial traction on the wrist with Chinese finger traps connected to weights, with manipulation by the caretaker of a combination of both. In most cases, a local anesthetic will be administered in the form of hematoma block with lidocaine between fracture fragments [7].

### Criteria for discontinuing or modifying allocated interventions {11b}

If patients allocated to the no closed reduction group develop neurovascular symptoms, the attending physician can opt to perform closed reduction regardless to relieve said symptoms.

### Strategies to improve adherence to interventions {11c}

Intervention will or will not be performed in the emergency department, so no additional strategies are required.

### Relevant concomitant care permitted or prohibited during the trial {11d}

Apart from the closed reduction, patients will receive standard care and have no trial specific limitations.

### Provisions for post-trial care {30}

Standard test subjects’ insurance has been taken out for all participants.

### Outcomes {12}

The primary outcome is the average of the pre-operative visual analog scale for pain (VAS) score, reported on a daily basis from the ED visit until surgery.

Secondary outcomes are wrist function measured with the Patient-Rated Wrist Evaluation (PRWE) score after 6 weeks and 3, 6, and 12 months, length of stay in the ED, type and quantity of used pain medication, patient satisfaction, quality of life, and complications. Furthermore, a cost-effectiveness analysis will be performed using the Medical Consumption Questionnaire (iMCQ) and Productivity Cost Questionnaire (iPCQ) [26, 27], and the ability to assess CT scans of unreduced fractures and reduced fractures will be compared.

### Participant timeline {13}

The participant timeline is shown in Table 1.

**Table 1:** Overview questionnaires

Name	Follow-up moment	Time spent per visit (minutes)	Time spent in total (minutes)
Listen, read and complete informed consent	ED	15	15
Pain diary	Every day until surgery	5	5 × 7–14* = 35–70
PRWE	6 weeks, 3 months, 6 months, 12 months	5	20
EQ-5D-5L	6 weeks, 3 months, 6 months, 12 months	5	20
ROM + grip strength	6 weeks, 3 months	1	2
Report used health resourced	6 weeks, 3 months, 6 months, 12 months	2	8
Report pain medication	6 weeks, 3 months, 6 months, 12 months	2	8
		**Total**	110–145

*Surgery usually takes place between 7 and 14 days

### Sample size {14}

**Fig. 1:**
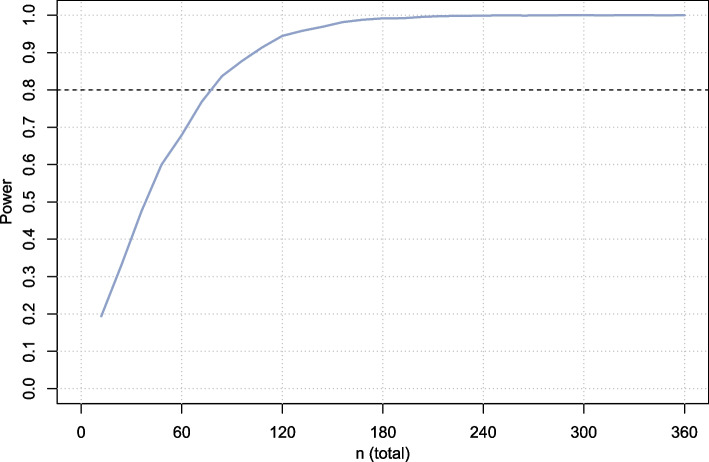
Power curve for simulation 1 assuming of balanced clusters (equal number of patients included per center)

The sample size calculation is based on our primary outcome parameter: the VAS score for pain reported on a daily basis from the visit to the ED until the day of surgery. To find out if the pre-operative pain scores of patients that are refrained from CR are comparable with the scores of the CR group, we will use a non-inferiority test. This requires a non-inferiority margin, which is the biggest difference between the two groups in favor of the CR group, without a statistical difference [28]. The European Medicines Agency advices to base the non-inferiority margin on a difference that is not clinically important [29]. Therefore, our non-inferiority margin is 50% of the minimal clinical important difference (MCID) of the VAS [28]. Based on a systematic review concerning the MCID of VAS scores, a MCID is a context-specific and methodological dependent value [30]. To the best of our knowledge, the MCID of the VAS score for DRFs is unknown. Therefore, we used the MCID of a study that included trauma patients with isolated acute extremity pain [31]. For the description of the MCID of the VAS score, the VAS scale is seen as a 100 mm line. With an MCID of 19.3 mm, our non-inferiority margin will be 9.7 mm (= 50%). As only Bird et al. provide a standard deviation (SD) around the MCID, we will use that value of 15 mm for our sample size calculation [24]. To correct for a cluster-effect we use an intra-cluster correlation (ICC) coefficient between the different hospitals of 0.06, which is generally reported in the literature for hospital processes. To calculate the required sample size, two simulations were run both assuming a fixed number of six clusters (hospitals). In the first simulation, an equal number of subjects per hospital was assumed. Hospitals were randomized to start with treatment A (3 centers) or B (3 centers) in a 1:1 fashion and crossed over after half of the patients were included. For total sample sizes varying from 12 to 502 with increments of 12, 5000 simulations were run for each sample size considered. In each simulation, random data was generated under the specifications described above. A linear mixed model was fitted with a random intercept for cluster and treatment group as fixed effect. A 95% confidence interval was calculated based on the t-distribution with degrees of freedom estimated by the Satterthwaite method. The power was calculated as the percentage of simulations in which the upper limit of the 95% confidence interval was smaller than the non-inferiority margin. From the first simulation, it followed that, using a two-sided alpha of 5%, *n* = 80 patients total would be required in total to demonstrate non-inferiority with a power of 80% (Fig. 1).

**Fig. 2:**
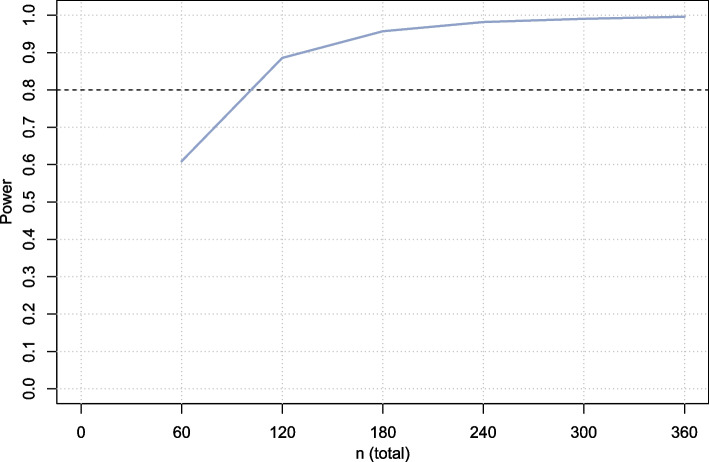
Power curve for simulation 2 assuming unbalanced clusters (unequal number of patients included per center) and adjustment for seasonal effects

However, in practice, unequal number of patients is expected to be included by each center. Also, seasonal effects should be accounted for in the analysis. Therefore, a second simulation was conducted in a similar way. The number of patients included per center was (roughly) based on the number of eligible patients in the past year (known for 4 hospitals) divided by 2 (assuming that 50% of eligible patients would participate). Inclusion rates for the two hospitals for which this was not known were set equal to the hospital with lowest inclusion rate, resulting in monthly inclusions of 13, 7, 7, 1, 1, 1. Seasonal effects were included by a monthly effect, which were assumed to follow a sine function with a period of 12 months. The SD of the seasonal effects over a 12-month period was set equal to the between cluster SD. The half period (*x* = *π*) was set equal to the moment of cross-over. For sample sizes varying from 60 (two months of inclusion) to 360 (12 months of inclusion), again 5000 simulations were run for each sample size considered. Linear mixed models were fitted including center as random effect and month and treatment as fixed effects. Using a two-sided alpha of 5%, *n* = 120 patients total (rounded to above) would be required in total to demonstrate non-inferiority with a power of 80% (Fig. 2). Accounting for a 10% loss to follow-up, a total of *n* = 134 patients will be included. It should be noted that in the simulations, it was assumed that only one VAS pain will be collected for each patient, while in reality multiple VAS scores (one per day) will be obtained during the period between the visit to the emergency department and the operation. An average VAS pain for each patient will be estimated by a multilevel linear mixed model using random intercept for patient nested within center. Including this aspect in the simulation studies was deemed too complicated, since no reliable estimates for the correlation between daily VAS pain scores in this setting are available. However, following the reasoning that multiple measures will lead to a more precise estimate than a single measure, it can be expected that in practice a higher power will be achieved compared to the simulations and including this is not strictly necessary (i.e., the simulations can be regarded as conservative in this respect).

In the Maasstad Hospital, approximately 590 adult patients are diagnosed with a DRF per year. According to Brogren and colleagues, approximately 350 (60%) will be displaced [5].

However, not all of these patients will have CR followed by surgical treatment. Assuming that 60% of all patients with a displaced DRF will meet the inclusion criteria, combined with a rejection rate of 30%, we estimate to require 13 months for inclusion in case of a single-center design. Assuming that the average amount of inclusions per month in the other participating centers equals the amount in the Maasstad Hospital, we can divide the required time by the amount of centers. With a total of six participating centers, we estimate to require 3 months to complete the inclusion. Combined with a follow-up time of 12 months and 3-month margin, the total required time for the clinical part of the trial will be 18 months.

### Recruitment {15}

Eligible patients at the ED, fit to the inclusion and exclusion criteria, will be informed about the study and invited to participate by the treating physician. The patient will also receive written information on the study and will have the opportunity to ask questions on before making the decision to participate or not. However, this needs to be done in a short period of time (approximately 30 min) because of the acute problem presentation and setting. If the patient fits the inclusion and exclusion criteria and signs the informed consent form, baseline information will be recorded.

## Assignment of interventions: allocation

### Sequence generation {16a}

Randomization at patient level will be challenging because of the 24/7 availability of the ED in all participating hospitals and therefore the 24/7 availability to include possible candidates. To overcome potential protocol violations and unnecessary loss of potential inclusions, randomization will take place as a cluster per hospital with a cross-over point halfway the needed inclusions per hospital (i.e., after 20 inclusions). This means that patients presenting at the same hospital will receive the same intervention, and halfway this will change to the other intervention (CR or withholding from CR). Using a simple script in R statistics, starting treatment will be randomly decided for each hospital, with guarantee that both treatments will occur three times.

### Concealment mechanism {16b}

Since this trial will not be blinded, there are no concealment mechanisms. We use a simple algorithm to randomly pick A (closed reduction) or B (no closed reduction) six times and allocate treatment by order of hospital recruitment.

### Implementation {16c}

The primary investigator generated the allocation sequence. Patients are enrolled by attending physicians in all participating hospitals. With the cluster randomization, every hospital will know beforehand what intervention to assign.

## Assignment of interventions: blinding

### Who will be blinded {17a}

This is an open-label study, and blinding is not possible.

### Procedure for unblinding if needed {17b}

The design is open label, so unblinding will not occur.

## Data collection and management

### Plans for assessment and collection of outcomes {18a}

The primary outcome is the average of the pre-operative visual analog scale for pain (VAS) score, reported on daily basis from the ED visit until operation. The VAS score is an easy, valid, and reliable tool for pain measurement that has been widely used [32]. The VAS score will be presented as a horizontal line with in which the complete left side is defined as “no pain,” and the complete right side is defined as “the most extreme pain possible.” The patients will be asked to mark the position that corresponds with their pain experience. The VAS score prior and after the plaster application will represent their current pain experience. During the ED visit, patients will receive a “pain diary,” containing multiple empty VAS forms. Patients will be asked to report their average VAS score at the end of every day as well as the worst pain of that day (the latter will be used for a secondary analysis). The average will be calculated by adding all pain scores and dividing by the number of days for each patient. Besides the pre-operative VAS scores, they will also register the post-operative VAS score at 6 weeks and at 3, 6, and 12 months. The following parameters will serve as secondary outcome measures.

Average pain per day, average highest pain, and highest pain per day will all be analyzed to identify possible patterns secondary to the primary outcome of average pain from day 1 until surgery. The post-operative Patient-Rated Wrist Evaluation (PRWE) score [33] after 6 weeks and 3, 6, and 12 months will be assessed. The PRWE is a 15-item questionnaire designed to measure wrist pain and disability in activities of daily living. The PRWE allows patients to rate their levels of wrist pain and disability from 0 to 10 and consists of 2 subscales:

- Pain subscale: contains 5 items each of which is further rated from 1 to 10. The maximum score in this section is 50 and minimum 0
- Function subscale: contains total 10 items which are further divided into 2 sections, i.e., specific activities (having 6 items) and usual activities (having 4 items). The maximum score in this section is 50 and minimum 0

The length of stay in the ED will be measured in minutes and concerns the entire time that a patient is present in the ED. This will be calculated by subtracting the registration time from the discharge time.

The type and quantity of painkillers used by the patients will be registered. In the ED, the pre-trauma painkiller use will be registered. Furthermore, the patient will be asked to keep records of their painkiller use in the diary on daily basis until operation.

Patient satisfactions with cosmetic result, functional result, and activity resumption will be determined at 12 months using a 10-point numerical rating scale (NRS), in which 0 implies extremely dissatisfied and 0 implies extremely satisfied.

Physical examination of range of motion (ROM) of the wrist will be measured actively with a goniometer in degrees. This will include palmar and dorsal flexion, ulnar and radial deviation, and pronation and supination of the wrist.

Grip strengths will be measured using a dynamometer as the mean of three measurements. Grips strength will be measured in kilograms.

The general quality of life will be measured with the EQ-5D-5L questionnaire. This questionnaire consists of five items, measuring at 5-point scales whether patients experience problems and to what extent, with regard to their mobility, self-care, daily activities, pain, and mood.

All described complications in the patient record will be reported, including, but not limited to, carpal tunnel syndrome (CTS), compartment syndrome, bleeding, infection, malunion, and revision surgery.

At the end of the study, we will assess whether CT scans of DRFs after CR are better to assess in pre-operative planning than CT scans of DRFs that did not have CR. Also, we will test inter-observer variability. Multiple experts will classify the fracture according to the AO classification on X-ray and CT scan. This will be blinded so the expert does not know which X-ray belongs to which CT scan.

The balance between costs and impact of refraining from CR in the ED will be assessed by performing a cost-effectiveness analysis. This will address the question whether or not withholding from CR is saving costs in ED. Cost-effectiveness will be calculated with the EQ-5D-5L, the iPCQ, and the iMCQ.

### Plans to promote participant retention and complete follow-up {18b}

Since participation to this study requires no extra hospital visits, we expect high follow-up percentage. Physical measurements will be taken at standard post op checkups at 6 weeks and 3 months; all other questionnaires will be sent by email or taken over the phone.

### Data management {19}

Data will be collected in Castor EDC. Data will be entered by study personnel from source documents kept in a locked storage. External data monitoring will take place at the start, half way, and at the end of the study.

### Confidentiality {27}

All participants will be given a site-specific number. As stated above, data will be stored in the save digital environment of Castor EDC, and physical forms will be stored in a locked storage. The data will only be accessible for the study personnel and an external monitor. All published data will be anonymized.

### Plans for collection, laboratory evaluation, and storage of biological specimens for genetic or molecular analysis in this trial/future use {33}

See the “ Additional consent provisions for collection and use of participant data and biological specimens {26b}” section; there will be no biological specimens collected, but the authors can tick a box on the informed consent form for the consent for the use of date for ancillary studies.

## Statistical methods

### Statistical methods for primary and secondary outcomes {20a}

Data for the primary outcome measure will be reported descriptively. Descriptive analysis will be performed in order to report outcome measures for the total population. Continuous data will be analyzed using an independent *t*-test or Mann–Whitney *U* test if not normally distributed. For continuous data, the mean and SD (parametric data) or the median and percentiles (non-parametric data) will be reported. For categorical data, numbers and frequencies will be reported. First, data will be reported for the entire study population. A subgroup analysis will be performed, discriminating between CR or withholding from CR. Differences in outcomes will be analyzed using the unpaired *T*-test in case of normally distributed data or the Mann–Whitney test in case of not normally distributed data. Separate analysis will be performed for average VAS score per patient and average VAS score per day to show both the overall average pain preoperatively and the daily trend towards day of surgery. This will show insight to possible effects on the average caused by patients waiting longer for their surgery. Descriptive analysis will be performed in order to report outcome measures for the total population (main analysis) and (if applicable) the subgroups as mentioned above. For continuous data, the mean and SD (parametric data) or the median and percentiles (non-parametric data) will be reported. For categorical data, numbers and frequencies will be reported. Given the expected low sample size, neither univariate nor multivariable analyses will be done. Trends in PRWE scores will be assessed using generalized linear mixed models calculating the marginal mean differences. Differences in complication rates and re-interventions will be analyzed using the chi-square test of Fisher’s exact test.

### Interim analyses {21b}

Not applicable. No interim analysis will be performed.

### Methods for additional analyses (e.g., subgroup analyses) {20b}

An economic evaluation will be conducted in accordance with the Dutch guidelines in which medical costs and loss of productivity costs will be considered. The time horizon will be 1 year to include all relevant costs and effects. Both a cost-effective (CEA) and a cost-utility (CUA) analysis will be performed. Direct intramural and extramural care costs will be calculated (i.e., radiographs, CT scan, casts, pain medication used, number of outpatient visits, surgical interventions, physiotherapy, hospital admission days). Indirect medical costs, such as productivity loss, will be calculated as well. Data on medical resources used will be collected from the iMTA Medical Consumption Questionnaire (iMCQ) [26]. Productivity costs will be registered in detail by the iMTA Productivity Costs Questionnaire (iPCQ) [27].

The primary economic outcome for the CEA is the costs per unit change in overall wrist function and closely relates to the clinical outcome measure. The primary economic outcome for the CUA is the costs per quality-adjusted life year (QALY).

A budget impact analysis (BIA) will be conducted from governmental and healthcare provider perspectives according to the guideline of Zorginstituut Netherlands, the Berenschot Leidraad Budget impact analyses, and the ISPOR recommendations from Sullivan et al. The observed healthcare costs per study group will be combined with the national incidence data for the target population to extrapolate the findings for the five coming calendar years, starting the first year after study closure, to estimate the financial consequences of wide-spread implantation of withholding from CR at the ED when there is an indication for surgery of the DR in the Dutch healthcare system.

Incidence data will be derived from past incidence data available from the Centraal Bureau voor de Statistiek (CBS), www.opendisdata.nl, and from MediRisk (the largest liability insurance company in the Netherlands).

The healthcare costs will reflect the index treatment, the density of complications, and follow-up treatments during the first 12 months of which the most will originate from hospitals, general practices, and physiotherapy practices.

### Methods in analysis to handle protocol non-adherence and any statistical methods to handle missing data {20c}

A linear mixed model will be used to handle missing data.

### Plans to give access to the full protocol, participant-level data, and statistical code {31c}

The datasets analyzed during the current study and statistical code are available from the corresponding author on reasonable request, as is the full protocol.

## Oversight and monitoring

### Composition of the coordinating center and trial steering committee {5d}

The trial is run by a group consisting of a principal investigator, a study coordinator, one local investigator for each participating hospital, a clinical statistician, and a research monitor; meetings occur weekly between principal and coordinating investigator and monthly between the study coordinator and local investigators. Progress will also be reported to the BeterKeten foundation, as they provided funding for this research. The nature of the treatment in our trial does not support input from patients during our study. Before creating this protocol, patients were surveyed on their experience on closed reduction to establish ground for this research. These results are not published separately.

### Composition of the data monitoring committee, its role and reporting structure {21a}

An external data monitor will be hired to test data quality and study procedure at the start, halfway point, and end of the study.

### Adverse event reporting and harms {22}

In accordance to Sect. 10, subsection 4, of the WMO, the sponsor will suspend the study if there is sufficient ground that continuation of the study will jeopardize subject health or safety. The sponsor will notify the accredited METC without undue delay of a temporary halt including the reason for such an action. The study will be suspended pending a further positive decision by the accredited METC. The investigator will ensure that all subjects are kept informed. All adverse events will be followed until they have abated or until a stable situation has been reached. Depending on the event, the follow-up may require additional tests or medical procedures as indicated and/or referral to the general physician or a medical specialist. SAEs need to be reported until the end of the study.

### Frequency and plans for auditing trial conduct {23}

The project management group, consisting of the principal investigator and study coordinator, will meet weekly, and the study coordinator will meet local investigators monthly to assess progress and trial conduct. Progress will also be reported to the ethics committee once a year and to BeterKeten foundation once a year, as they provided funding for this research.

### Plans for communicating important protocol amendments to relevant parties (e.g., trial participants, ethical committees) {25}

Amendments are changes made to the research after a favorable opinion by the accredited METC has been given. All substantial amendments will be notified to the METC that gave a favorable opinion.

Non-substantial amendments will not be notified to the accredited METC but will be recorded and filed by the sponsor. Examples of non-substantial amendments are typing errors and administrative changes like changes in names, telephone numbers, and other contact details of involved persons mentioned in the submitted study documentation.

### Dissemination plans {31a}

Primary outcomes will be published in several research articles. We aim to also present our results at both national and international conferences for orthopedic trauma surgeons, hand surgeons, and all emergency department personnel as well as through our sponsor. The principal investigator is a member of the guideline committee and will assist in implementing our findings in the new guidelines.

## Discussion

The main practical issue we focus on during this trial is the decision of the attending trauma surgeon whether or not the fracture requires surgical fixation. We trust all participating physicians adhere to the national guidelines, but practice shows at least some amount of inter-observer variability, with several cases of initial conservative treatment, with the intention of avoiding surgery. To monitor this, all exclusions that were eligible for inclusion according to the stated criteria will be kept track of, including the reason for exclusion.

## Trial status

The RECORDED trial with protocol version 2.0 dating February 10, 2023, was accepted by the ethics committee on January 27, 2023. The first inclusion was on June 23, 2023, and recruitment is expected to be completed in April 2024.

## Data Availability

The principal and the coordinating investigator will have direct access to the final trial dataset as well as the statistician. The datasets used and/or analyzed for this study will be available from the corresponding author on reasonable request.

## Abbreviations

ER: Emergency department
DRF: Distal radius fracture
CR: Closed reduction
DRUJ: Distal radioulnar joint
ROM: Range of motion
VAS: Visual analog scale
NRS: Numerical rating scale
PRWHE: Patient-Rated Wrist and Hand Questionnaire
MCID: Minimal clinically important difference
CTS: Carpal tunnel syndrome
METC: Medisch Etische Toetsings Commissie (ethics committee)
